# Comparing the outcomes of the technology‐enhanced learning of sectional anatomy with and without preceding demonstrations: A study about the worked‐example effect in anatomy education

**DOI:** 10.1002/ase.70267

**Published:** 2026-05-25

**Authors:** Guilherme Reis Borges Coelho Fonseca, George Lim Tipoe, Fraide Augustin Ganotice

**Affiliations:** ^1^ School of Biomedical Sciences, Li Ka Shing Faculty of Medicine The University of Hong Kong Hong Kong China; ^2^ Bau Institute of Medical and Health Sciences Education, Li Ka Shing Faculty of Medicine The University of Hong Kong Hong Kong China

**Keywords:** anatomy, cognitive load theory, cognitive theory of multimedia learning, instructional efficiency, self‐determination theory, technology‐enhanced learning, usability, worked example

## Abstract

The examination of anatomical cross‐sections can facilitate the understanding of spatial and clinical anatomy. Visualization technologies, with dynamic and interactive displays, can be used to learn sectional anatomy in a mostly self‐directed manner. Nevertheless, this study hypothesized that the technology‐enhanced learning of sectional anatomy is improved when initiated with explicit anatomical demonstrations—worked examples, as defined in Cognitive Load Theory (CLT) and Cognitive Theory of Multimedia Learning (CTML). Two learning experiments were conducted with Year 1 (*N* = 110) and Year 2 (*N* = 59) undergraduate medical students who had to learn the sectional anatomy of the chest and abdomen, respectively. In both experiments, participants were first stratified according to gender and then randomly allocated into Groups A or B. Group A (control) used the website IMAIOS.com in a self‐directed manner to learn sectional anatomy. Group B (worked example) watched a demonstration of the sectional anatomy to be learned before using the website. Both groups had 35 min to complete the activity. In both experiments, several measurements (knowledge, mental effort, instructional efficiency, and types of cognitive load) showed that learning was more effective and efficient in Group B than in Group A. No intergroup differences were found in student motivation or in perceptions of IMAIOS.com usability. As predicted in CLT and CTML, worked examples improved the technology‐enhanced learning of sectional anatomy. While advanced students may benefit from more self‐directed learning approaches, novice learners may require preceding demonstrations to guide their attention and help organize their thinking process.

## INTRODUCTION

The examination of anatomical cross‐sections can promote the comprehension of imaging anatomy, spatial anatomy, and clinical anatomy. For instance, in some laboratory sessions at the University of Hong Kong, dental students have to examine potted cross‐sections of the head and neck to (1) find structures that are annotated in corresponding computed tomography (CT) and magnetic resonance (MR) images; (2) locate and outline the tissue spaces of the head and neck that can transmit odontogenic infections; and (3) indicate the approximate course of needles in various nerve blocks. Afterward, students receive feedback by covering the cross‐sections with annotated transparencies. Similarly, several visualization technologies, with dynamic and interactive anatomical images, have been increasingly used for the instruction of sectional anatomy. For instance, DICOM viewers^1^, ^2^, ^3^; virtual dissection tables^4^, ^5^, ^6^; digital platforms^7^, ^8^, ^9^; and extended reality systems^10^, ^11^, ^12^ have been used to show not only 3D whole anatomy but also 2D sectional anatomy, via CT images, MR images, and even cryosections derived from Visible Human Projects.^13^, ^14^, ^15^

Despite the potential of these educational technologies, students still perceive learning 2D sectional anatomy as challenging.^5^, ^16^ Research has shown that anatomy learners exhibit increased mental effort and reduced accuracy in locating anatomical structures in 2D sectional images versus in 3D representations.^17^, ^18^ Novice learners of anatomy, with insufficient anatomical knowledge and spatial abilities, may find it difficult to recognize 3D structures from 2D cross‐sections, and vice versa.^3^ Thus, when engaging with, for example, CT images, students may ask “How do I know what I am looking at in the CT scan?” or “Where do I start my analysis?” even when such images are labeled.^3^ These challenges could be related to the spatial abilities of learners, to the time allocated for anatomy teaching and learning, to the instructional resources used, and also to the instructional methods.

Principles of instructional design prescribed in learning theories, such as the Cognitive Load Theory (CLT)^19^, ^20^ and the closely related Cognitive Theory of Multimedia Learning (CTML),^21^, ^22^ can be leveraged to adjust the technology‐enhanced instruction of spatial anatomy, namely sectional anatomy, to benefit both low and high spatial ability students. CLT and CTML have been built on decades of scholarship in educational and cognitive psychology.^23^, ^24^, ^25^ These theories use the knowledge of how people learn, think, and solve problems to propose how instructional activities and materials should be designed.^20^, ^26^, ^27^, ^28^ A concept central to CLT and CTML is that the working memory of a learner—meaning, the learner's ability to maintain and manipulate information in active attention—has a finite capacity.^29^, ^30^, ^31^, ^32^ While learning, the working memory capacity is used and potentially depleted, as more cognitive load is experienced by the learner. Cognitive load is a multidimensional construct composed of three elements.^19^, ^20^, ^27^ First, intrinsic cognitive load relates to the inherent complexity of the learning contents and to the degree of students' prior expertise with said information. It tends to be higher when the learning contents are difficult and when learners have little to no prior knowledge of the learning contents. Second, germane cognitive load expresses the conscious and effortful internal process of organizing and integrating new information. It tends to be higher when learners are more mentally engaged with the learning content. Third and finally, extraneous cognitive load represents the mental resources that are wasted in mental tasks that do not facilitate the achievement of learning objectives. It is caused by poor instructional design. Extraneous cognitive load hinders effective learning and should always be reduced. Overall, CLT and CTML researchers have been continuously presenting empirically supported teaching strategies aimed at making learning more effective and efficient by reducing extraneous cognitive load, managing intrinsic cognitive load, and optimizing the germane cognitive load of learners.^20^, ^26^, ^27^, ^28^, ^31^, ^33^

For instance, a worked example is a form of explicit instruction, often used in mathematics, that has been extensively studied within the fields of CLT and CTML.^34^, ^35^ In contrast to more exploratory learning activities, a worked example immediately provides the solution to the learning problem and the steps to understand it.^27^, ^34^, ^35^, ^36^ This facilitates initial knowledge acquisition and transfer performance as it reduces the extraneous cognitive load of novice learners who otherwise would need to invest their working memory in developing their own problem‐solving strategies.^31^, ^33^ Although not usually mentioned as such, worked examples are regularly conducted in anatomy teaching. For instance, in dissection sessions at the University of Hong Kong, medical students watch brief instructional videos that show not only the steps of the dissection to be performed, but also the expected normal size, shape, and location of the structures to be identified and isolated. Parallelly, in prosection sessions, dental students observe brief and outcome‐focused teacher demonstrations that provide immediate transferable knowledge about how to examine prosections of the head and identify structures in a systematic fashion. In both cases, students are exposed to learning content delivered in a direct, methodical, and explicit audiovisual manner before having to purposefully explore anatomical specimens by themselves. Ergo, it can be reasoned that a similar type of worked example—an anatomical demonstration—can also be useful in the technology‐enhanced instruction of sectional anatomy.

### Objectives and hypotheses of the study

The aim of this study was to investigate whether the integration of a worked example, provided in the form of a prerecorded audiovisual anatomical demonstration, improves the technology‐enhanced learning of sectional anatomy. Three main hypotheses were developed based on the CLT and CTML:

The integration of worked examples in the technology‐enhanced learning of sectional anatomy increases knowledge gains.

The integration of worked examples in the technology‐enhanced learning of sectional anatomy lowers extraneous cognitive load.

The integration of worked examples in the technology‐enhanced learning of sectional anatomy increases instructional efficiency.

Moreover, this study aimed to explore whether students' motivation and perceptions of ed‐tech usability influenced, or were influenced by, the tested instructional conditions.

## MATERIALS AND METHODS

**FIGURE 1:**
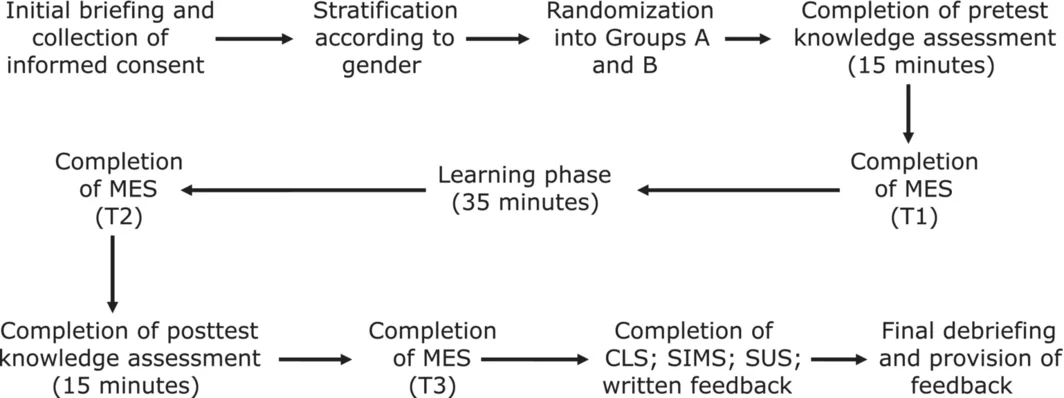
Study design. These sessions were conducted in March 2025 with Year 1 medical students who had to learn the sectional anatomy of the chest. Later, in April 2025, sessions were conducted with Year 2 medical students who had to learn the sectional anatomy of the abdomen. Participants were briefed about the basic structure and purpose of the assessments and scales they had to complete. CLS, cognitive load scale. MES, mental effort scale. SIMS, situational motivation scale. SUS, system usability scale.

This study employed a longitudinal pretest‐posttest design (Figure 1). It encompassed two experimental learning sessions with two samples of students. Ethical approval was obtained from the Institutional Review Board of the University of Hong Kong (reference UW 21‐432). All participants provided informed consent.

### Sampling procedures

Year 1 and Year 2 undergraduate medical students from the Faculty of Medicine of the University of Hong Kong were invited to participate in this study through e‐mail and social media adverts. This study aimed to recruit the largest sample possible. Year 1 students participated in an experimental learning session where they had to learn the sectional anatomy of the chest. Year 2 students participated in a different session where they had to learn the sectional anatomy of the abdomen. Both sessions took place in the Technology‐Enriched Learning Mezzanine of the Yu Chun Keung Medical Library and had to be repeated multiple times to accommodate all participants.

### Experimental procedures

In both the Year 1 and Year 2 experimental learning sessions, participants were first stratified according to gender. This stratification was conducted because of the possible impact of gender on spatial abilities and the subsequent effect on spatial anatomy learning.^37^, ^38^ Participants from each stratum were randomly allocated, via a simple paper lottery, into Groups A and B. Participants of both groups were tasked to, individually and sequentially (1) watch a prerecorded instructional video; and (2) use the paid version of the IMAIOS.com eLearning website to identify specific anatomical structures in transverse CT images that were dynamic (i.e., scrollable) and interactive (i.e., with selectable labels). The total time‐on‐topic provided for both groups was 35 min.

#### Group A (control): Shorter video without anatomical demonstration. More time for self‐directed learning

Participants allocated to Group A watched a shorter video (2:35 in Year 1 session; 3:04 in Year 2 session) before using the IMAIOS.com platform by themselves. Hence, participants spent most of the 35 min exploring the CT images in a self‐directed manner. The video shown to Group A included only a brief introduction to the learning activity; an explanation of basic concepts required for CT image orientation and interpretation; and suggestions on how to interact with the IMAIOS.com interface to achieve the learning outcomes.

#### Group B (worked example): Longer video with anatomical demonstration. Less time for self‐directed learning

Conversely, participants allocated to Group B watched a longer video (18:10 in Year 1 session; 23:29 in Year 2 session). Thus, participants of this group had relatively less time to explore the CT images in IMAIOS.com by themselves. Their video included the same briefing presented in the Group A video, plus an audiovisual demonstration of the sectional anatomy to be learned. The demonstrations comprised the identification of the required structures in the CT images of IMAIOS.com and explanations about their anatomical spatial relationships.

#### Learning outcomes and materials

Overall, Year 1 participants had to identify 35 structures of the chest. Year 2 participants had to identify 36 abdominal structures. Thus, printed lists with the thoracic/abdominal structures to be identified were given to participants together with two supporting Netter illustrations. The lists were created by surveying textbooks in sectional and radiological anatomy^39^, ^40^, ^41^, ^42^, ^43^ and by evaluating the needs of the participants. At the beginning of this study, Year 1 medical students had completed the dissection of the chest as part of their Cardiopulmonary and Renal Systems learning block. Their Year 2 seniors had completed the dissection of the abdomen as part of their Gastrointestinal System learning block. Both cohorts of students had also attended introductory lectures in thoracic and abdominal radiology.

The instructional videos, including the anatomical demonstrations, were created with the sound and screen recording functions of Panopto.com, an online video platform. Moreover, the dynamic and interactive transverse CT images in the interface of IMAIOS.com were presented alongside static images of the scanned body that functioned as cross‐references. Year 1 participants accessed https://www.imaios.com/en/e‐anatomy/thorax/ct‐axial‐chest (as retrieved in March 2025) to identify the structures of the chest. Year 2 participants accessed https://www.imaios.com/en/e‐anatomy/abdomen‐and‐pelvis/ct‐axial‐male‐abdomen‐and‐pelvis (as retrieved in April 2025) to identify the structures of the abdomen. The website had been successfully used in a previous exploratory study.^44^

### Measurements

The variables assessed in this study were (1) anatomy knowledge; (2) mental effort and instructional efficiency; (3) types of cognitive load; (4) situational motivation and self‐determination; (5) usability of educational technology; and (6) student perceptions of the learning activity.

#### Anatomy knowledge

Participants in each learning session completed an anatomy test immediately before and after the learning activity to measure knowledge gains in sectional anatomy of the chest (Year 1 experiment) or abdomen (Year 2 experiment). Therefore, two anatomy tests were constructed to be aligned with the learning outcomes of the chest‐focused/abdomen‐focused learning activities. The two tests consisted of 20 multiple‐choice question items that required the identification of anatomical structures in transverse CT images of the chest/abdomen.^39^, ^40^, ^41^, ^42^, ^43^ The order of the items was changed in the posttests to reduce the possible influence of pretest execution on posttest performance.^44^, ^45^, ^46^ Participants had 15 min to complete each test and received 1 mark for each correct answer and 0 marks for each incorrect answer. Pretest and posttest knowledge scores were converted into percentages and used to calculate absolute learning gains (*g*_abs_) with the formula below.^47^, ^48^

$$g_{\mathrm{abs}}=P_{\text{posttest}}\left(\%\right)-P_{\text{pretest}}\left(\%\right)$$

where *g*_abs_ is absolute learning gain, and *P* is mean score.

It is important to note that the calculation of absolute learning gains does not account for the fact that students with low pretest scores have more to gain than students with high pretest scores.^49^ Essentially, a strong negative correlation exists between students' absolute learning gains and pretest scores.^49^ This happens because test scores have an upper limit (20 marks in the current study). Thus, normalized learning gains (*g*_*i*_) were calculated with the formula below.^47^, ^48^, ^49^ As defined by Hake,^49^ *g*_*i*_ < 0.3 was considered low; 0.3 ≤ *g*_*i*_ < 0.7 was considered medium; *g*_*i*_ ≥ 0.7 was considered high.

$$g_{i}=\frac{\left[P_{\text{posttest}}\left(\%\right)-P_{\text{pretest}}\left(\%\right)\right]}{\left[100\%-P_{\text{pretest}}\left(\%\right)\right]}$$

where *g*_*i*_ is normalized learning gain, and *P* is mean score.

The content validity of the tests was expressed via a Content Validity Index of 1, which was obtained after analysis by three anatomy lecturers.^50^ The tests were piloted with seven Year 3 and Year 4 medical students. The chest‐focused and abdomen‐focused tests revealed Cronbach's alphas of 0.60 and 0.64, respectively, which were considered acceptable.^51^

#### Mental effort and instructional efficiency

Participants used a 9‐point Likert Mental Effort Scale (MES) to rate, on a scale ranging from 1 (*very, very low mental effort*) to 9 (*very, very high mental effort*), the mental effort required during the pretest phase, learning phase, and posttest phase.^52^ The construct “mental effort” has been used as an indicator of total cognitive load.^53^, ^54^ Accordingly, the MES has been used before to measure the total cognitive load experienced by learners of anatomy.^55^, ^56^, ^57^, ^58^, ^59^

The results of the MES at the learning phase and posttest phase were combined with the results of the posttest knowledge assessment to calculate different indices of instructional efficiency.^60^, ^61^, ^62^ The overarching concept of instructional efficiency is concerned with the relationship between the mental effort required to complete a learning activity or related test and the corresponding test performance.^62^ In essence, a highly efficient instructional activity leads to the achievement of high academic performance with low mental effort. On the other hand, low instructional efficiency relates to low performance and high effort.

First, the individual scores of mental effort at the learning phase, mental effort at the posttest phase, and posttest knowledge were converted into *z*‐scores (i.e., standardized values with a mean of 0 and a standard deviation of 1) by using the formula below.^60^

$$Z=\frac{r-M}{\mathrm{SD}}$$

where *Z* is *z*‐score, *r* is individual raw score, *M* is mean score of the sample, and SD is standard deviation of the sample.

Second, two 2‐dimensional indices of relative instructional efficiency were calculated with the formulas below.^62^

$$2D\;\text{Efficiency}\;\left(L\right)=\frac{P-\mathrm{EL}}{\sqrt{2}}$$

where *P* is *z*‐score of posttest knowledge, *EL* is *z*‐score of mental effort at the learning phase.

$$2D\;\text{Efficiency}\;\left(T\right)=\frac{P-\mathrm{ET}}{\sqrt{2}}$$

where *P* is *z*‐score of posttest knowledge, *ET* is *z*‐score of mental effort at the posttest phase.

Third, one 3‐dimensional index of relative instructional efficiency was calculated as follows^61^:

$$3D\;\text{Efficiency}=\frac{P-\mathrm{EL}-\mathrm{ET}}{\sqrt{3}}$$

where *P* is *z*‐score of posttest knowledge, *EL* is *z*‐score of mental effort at the learning phase, and *ET* is *z*‐score of mental effort at the posttest phase.

#### Types of cognitive load

Participants also used an adapted version of the Cognitive Load Scale (CLS) to measure the different types of cognitive load experienced in the learning activity.^63^ The CLS is a valid and reliable instrument^63^, ^64^, ^65^ composed of 10 items organized into three subscales that quantify intrinsic cognitive load, extraneous cognitive load, and germane cognitive load. Participants responded to each item with a scale ranging from 0 (*not at all the case*) to 10 (*completely the case*).

Even though the CLS has been used before in anatomy educational research,^66^, ^67^ this multidimensional instrument was originally developed to assess the cognitive load of students attending statistics and language lectures.^63^, ^65^ Therefore, the wording of the items was changed to fit the setting of this study.^68^ Subsequently, three anatomy lecturers conducted a content validity check, via group discussion, to ensure that the adapted CLS was adequate for the current study. The content validity of the adapted CLS was expressed with a Content Validity Index of 1.^50^

#### Situational motivation and self‐determination

Participants completed a previously adapted version of the Situational Motivation Scale (SIMS) to measure motivation.^69^ The SIMS is a valid and reliable instrument^70^ comprised of 16 items organized into four subscales that assess intrinsic motivation; extrinsic motivation (specifically identified regulation and external regulation); and amotivation.^70^ Participants responded to each item with a scale ranging from 1 (*corresponds not at all*) to 7 (*corresponds exactly*). This popular instrument has been used before to measure the situational motivation of anatomy students.^71^, ^72^

According to Self‐Determination Theory,^73^, ^74^, ^75^ the different types of motivation that regulate human behavior are arranged along a continuum based on their degree of autonomy and underlying locus of causality, from internal (intrinsic motivation), to external (extrinsic motivation), to impersonal (amotivation). Thus, measures of each type of motivation were weighted, according to their position in this self‐determination continuum, and used to calculate a self‐determination index with the formula: 2*(intrinsic motivation) + 1*(identified regulation) − 1*(external regulation) − 2*(amotivation).^76^, ^77^ Educational research has recurrently shown a positive association between more internally localized motivation, and thus self‐determination, with academic achievement.^78^

#### Usability of educational technology

Participants completed the System Usability Scale (SUS) to measure the usability of the IMAIOS.com website.^79^ The SUS is composed of 10 items that were answered with a scale ranging from 1 (*strongly disagree*) to 5 (*strongly agree*). The item scores were used to compute a final SUS mark that ranged from 0 to 100.^79^, ^80^ A final SUS mark of 68 was used as a benchmark of usability.^81^, ^82^ Overall, the SUS is an extremely popular standardized measurement of usability with robust evidence supporting its validity, reliability, and sensitivity.^81^ Furthermore, the SUS has been used before to assess the usability of educational technologies used in anatomy teaching and learning.^44^, ^55^, ^83^

#### Student perceptions of the learning activity

Finally, participants were asked to write their opinions regarding the learning session. They were prompted with the following open question: “*What are your thoughts about this experimental learning activity? (e.g., What did you like? What did you dislike? What could have been improved? What are your comments and suggestions?).”* Qualitative data were collected to complement the quantitative data.^84^

### Analysis of results

Initial sorting of raw quantitative data (knowledge test results, MES results, CLS results, SIMS results, SUS results) and calculation of relevant indices were conducted in Microsoft® Excel®. Descriptive and inferential analyses were conducted in IBM® SPSS® Statistics Version 28.^85^ Shapiro–Wilk tests were used to assess the distribution of data in each group across all parameters. Paired *t*‐tests, or Wilcoxon signed‐rank tests, were used to compare pretest knowledge and posttest knowledge in each group. This was conducted to assess whether significant knowledge gains were achieved by Groups A and B. Cohen's *d*, or effect size estimate *r*, was used to determine effect sizes. Independent *t*‐tests, or Mann–Whitney *U* tests, were used to compare pretest knowledge, posttest knowledge, MES scores, indices of instructional efficiency, CLS subscale scores, SIMS subscale scores, self‐determination index, and SUS scores between Groups A and B. This was conducted to determine whether significant differences existed between groups. A level of significance of 0.05 was determined for all statistical tests. Furthermore, the internal consistency of the anatomy tests, subscales of CLS, subscales of SIMS, and SUS was assessed with Cronbach's alpha. The written feedback was transcribed to Microsoft® Word® for coding and thematic analysis.^86^, ^87^ The analysis was guided by the question: What were the students' thoughts about the learning session?

## RESULTS

**FIGURE 2:**
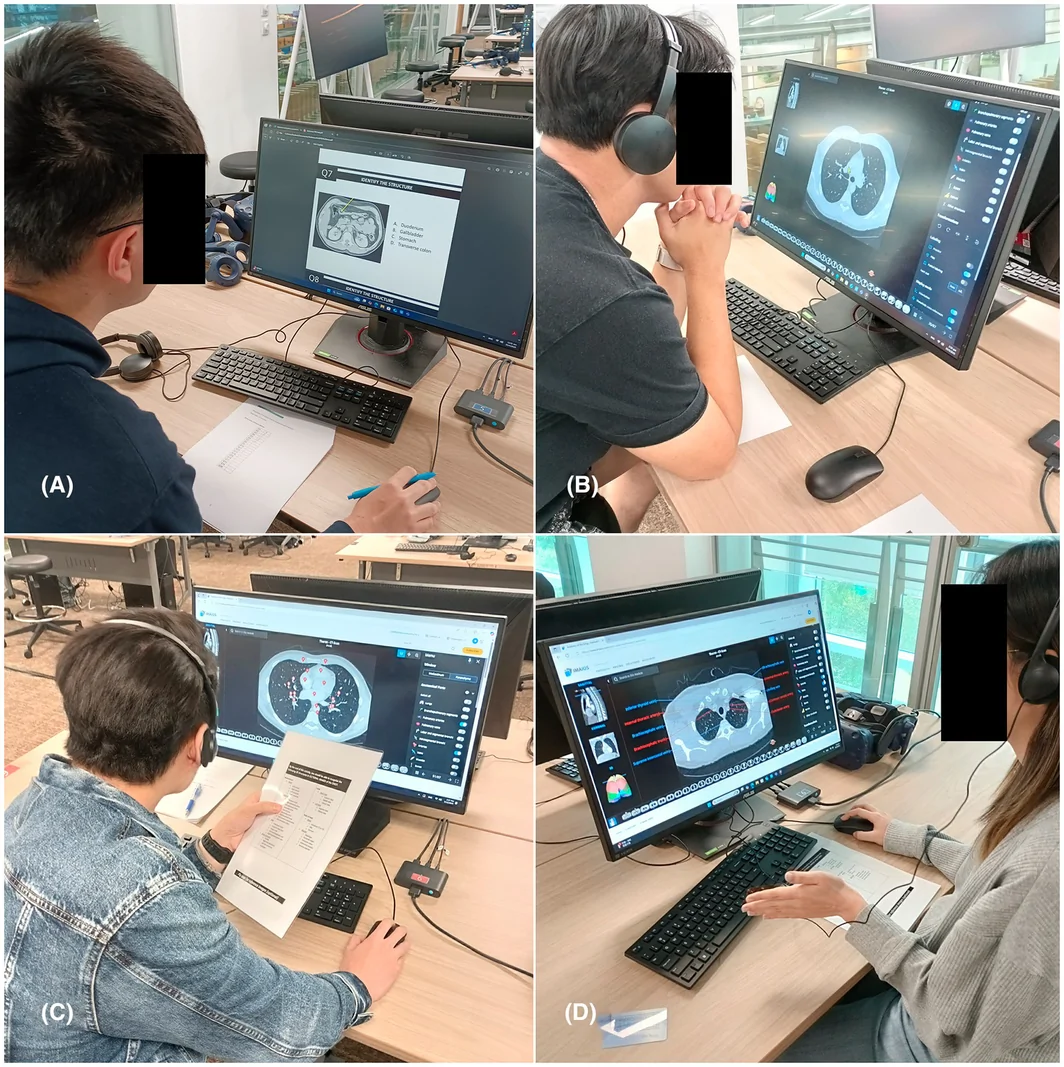
(A) Year 2 participant completing the sectional anatomy pretest. The order of the test items was changed for the posttest assessment. (B) Year 1 participant of Group B watching the prerecorded video with the anatomical demonstration. The videos watched by Group A, in both Year 1 and Year 2 sessions, were considerably shorter as they did not include an anatomical demonstration. (C) Year 1 participant using tools of interactivity in the IMAIOS.com interface while holding the list of structures to identify. (D) Year 1 participant using selected labels in the IMAIOS.com interface.

In total, 169 undergraduate medical students participated in this study (Figure 2). Specifically, 110 Year 1 students, with a mean age of 19.08 years old, participated in the chest‐focused learning session (Table 1). In addition, 59 Year 2 students, with a mean age of 20.37 years old, participated in the abdomen‐focused learning session (Table 1). All participants completed the experiment, including all quantitative assessments.

**TABLE 1:** Demographic information of the samples and summary of results in both Year 1 and Year 2 experiments.

		Year 1 sample (*N* = 110)		Year 2 sample (*N* = 59)	
		Group A (*n* = 54)	Group B (*n* = 56)	Group A (*n* = 28)	Group B (*n* = 31)
Demographic information					
Gender	Female	24	24	14	15
	Male	30	32	14	16
Prior use of IMAIOS.com	Yes	1	1	5	5
	No	53	55	23	26
Summary of results					
Anatomy knowledge (anatomy test)	Pretest	45.74	46.96	79.29	73.71
	Posttest	**74.35**	**83.84**	84.82	89.03
	Absolute gain	28.61	36.88	5.53	15.32
	Normalized gain	0.53 (medium)	0.70 (high)	0.27 (low)	0.58 (medium)
Mental effort and instructional efficiency (MES)	Pretest	7.39	7.45	6.61	6.65
	Learning	6.72	6.23	6.32	5.97
	Posttest	5.24	5.45	5.39	5.13
	2D Efficiency (L) index	**−0.37**	**0.36**	−0.21	0.19
	2D Efficiency (T) index	**−0.20**	**0.20**	−0.19	0.18
	3D Efficiency index	**−0.26**	**0.25**	−0.23	0.21
Types of cognitive load (CLS)	Intrinsic load	6.52	5.76	6.02	5.67
	Extraneous load	**1.43**	**0.85**	**1.24**	**0.75**
	Germane load	8.25	8.51	7.81	8.22
Situational motivation and self‐determination (SIMS)	Intrinsic motivation	4.80	5.03	4.68	4.69
	Identified regulation	6.19	6.17	5.97	6.15
	External regulation	2.94	2.93	2.84	2.89
	Amotivation	1.78	1.66	1.67	1.78
	Self‐determination index	9.27	9.98	9.15	9.07
Usability of educational technology (SUS)		77.96	78.79	80.54	81.94

*Note*: Significant intergroup differences are in bold (*p* < 0.05).

### Anatomy knowledge

**FIGURE 3:**
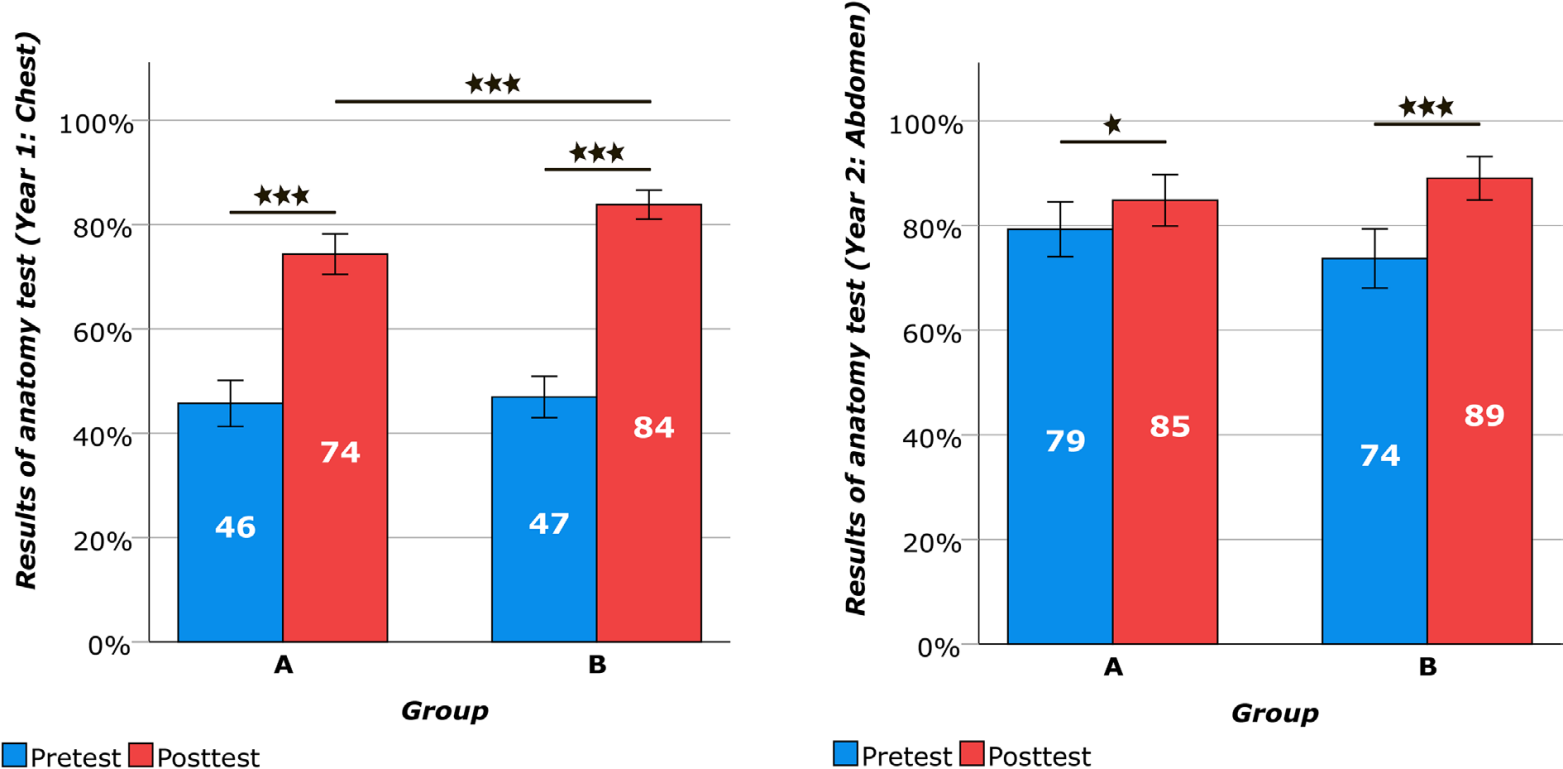
**Left graph:** Results of anatomy tests in Year 1 experiment. At pretest, the knowledge scores of Group A (mean = 45.74; SD = 16.24) were lower (*U* = 1630.000; *p* = 0.478) than Group B (mean = 46.96; SD = 14.76). At posttest, the knowledge scores of Group A (mean = 74.35; SD = 14.24) were significantly lower (*U* = 2119.000; *p* < 0.001) than Group B (mean = 83.84; SD = 10.31). Posttest knowledge scores were significantly greater than the pretest scores in both Group A (*W* = 1422.500; *p* < 0.001; *r* = 0.60) and Group B (*W* = 1485.000; *p* < 0.001; *r* = 0.61). Group A (*g*_abs_ = 28.61; *g*_*i*_ = 0.53) acquired less knowledge than Group B (*g*_abs_ = 36.88; *g*_*i*_ = 0.70). The pretest and posttest knowledge assessments exhibited a Cronbach's alpha of 0.56 and 0.63, respectively. Combined, they showed a Cronbach's alpha of 0.81. Whiskers indicate the 95% confidence interval. **Right graph:** Results of anatomy tests in Year 2 experiment. At pretest, the knowledge scores of Group A (mean = 79.29; SD = 13.52) were greater (*U* = 335.500; *p* = 0.131) than Group B (mean = 73.71; SD = 15.44). At posttest, the knowledge scores of Group A (mean = 84.82; SD = 12.66) were lower (*U* = 528.500; *p* = 0.145) than Group B (mean = 89.03; SD = 11.36). Posttest knowledge scores were significantly greater than the pretest scores in both Group A (*W* = 209.500; *p* = 0.028; *r* = 0.29) and Group B (*W* = 406.000; *p* < 0.001; *r* = 0.59). Group A (*g*_abs_ = 5.53; *g*_*i*_ = 0.27) acquired less knowledge than Group B (*g*_abs_ = 15.32; *g*_*i*_ = 0.58). The pretest and posttest knowledge assessments exhibited a Cronbach's alpha of 0.68 and 0.69, respectively. Combined, they showed a Cronbach's alpha of 0.72. Whiskers indicate the 95% confidence interval.

In both experiments, the anatomy tests showed that Groups A and B acquired significant knowledge gains in sectional anatomy. In both experiments, Group B gained more knowledge than Group A (Figure 3).

### Mental effort and instructional efficiency

**FIGURE 4:**
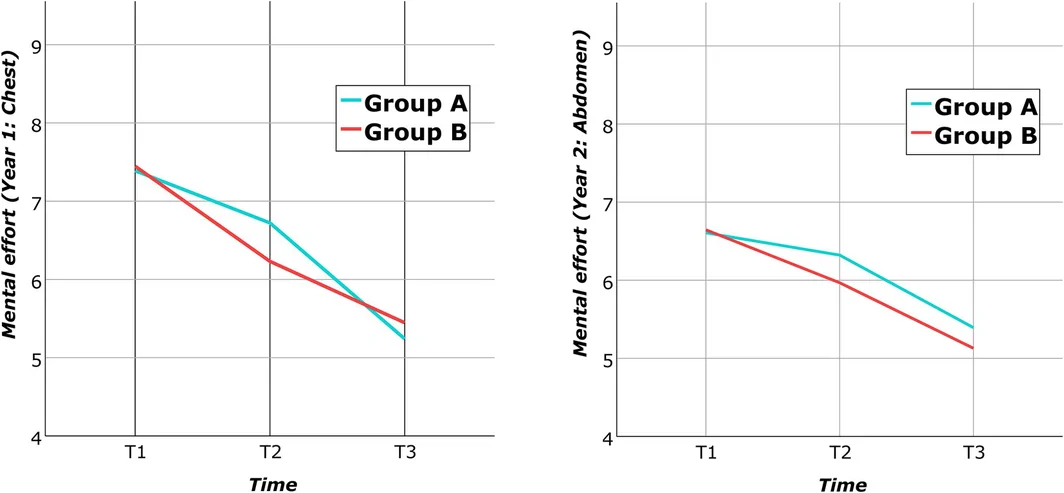
**Left graph:** Results of MES in Year 1 experiment. The mean MES scores of Group A at pretest, learning phase, and posttest were 7.39 (SD = 0.98), 6.72 (SD = 1.56), and 5.24 (SD = 1.36), respectively. The mean MES scores of Group B at pretest, learning phase, and posttest were 7.45 (SD = 1.16), 6.23 (SD = 1.50), and 5.45 (SD = 1.37), respectively. There were no significant intergroup differences in pretest phase (*U* = 1595.000; *p* = 0.602), learning phase (*U* = 1200.000; *p* = 0.057), and posttest phase (*U* = 1600.000; *p* = 0.588). T1, pretest phase. T2, learning phase. T3, posttest phase. **Right graph:** Results of the MES test in Year 2 experiment. The mean MES scores of Group A at pretest, learning phase, and posttest were 6.61 (SD = 1.07), 6.32 (SD = 1.52), and 5.39 (SD = 1.45), respectively. The mean MES scores of Group B at pretest, learning phase, and posttest were 6.65 (SD = 1.36), 5.97 (SD = 1.58), and 5.13 (SD = 1.57), respectively. There were no significant intergroup differences in pretest phase (*U* = 443.500; *p* = 0.882), learning phase (*t* = 0.875; *p* = 0.193), and posttest phase (*t* = 0.670; *p* = 0.253). T1, pretest phase. T2, learning phase. T3, posttest phase.

**FIGURE 5:**
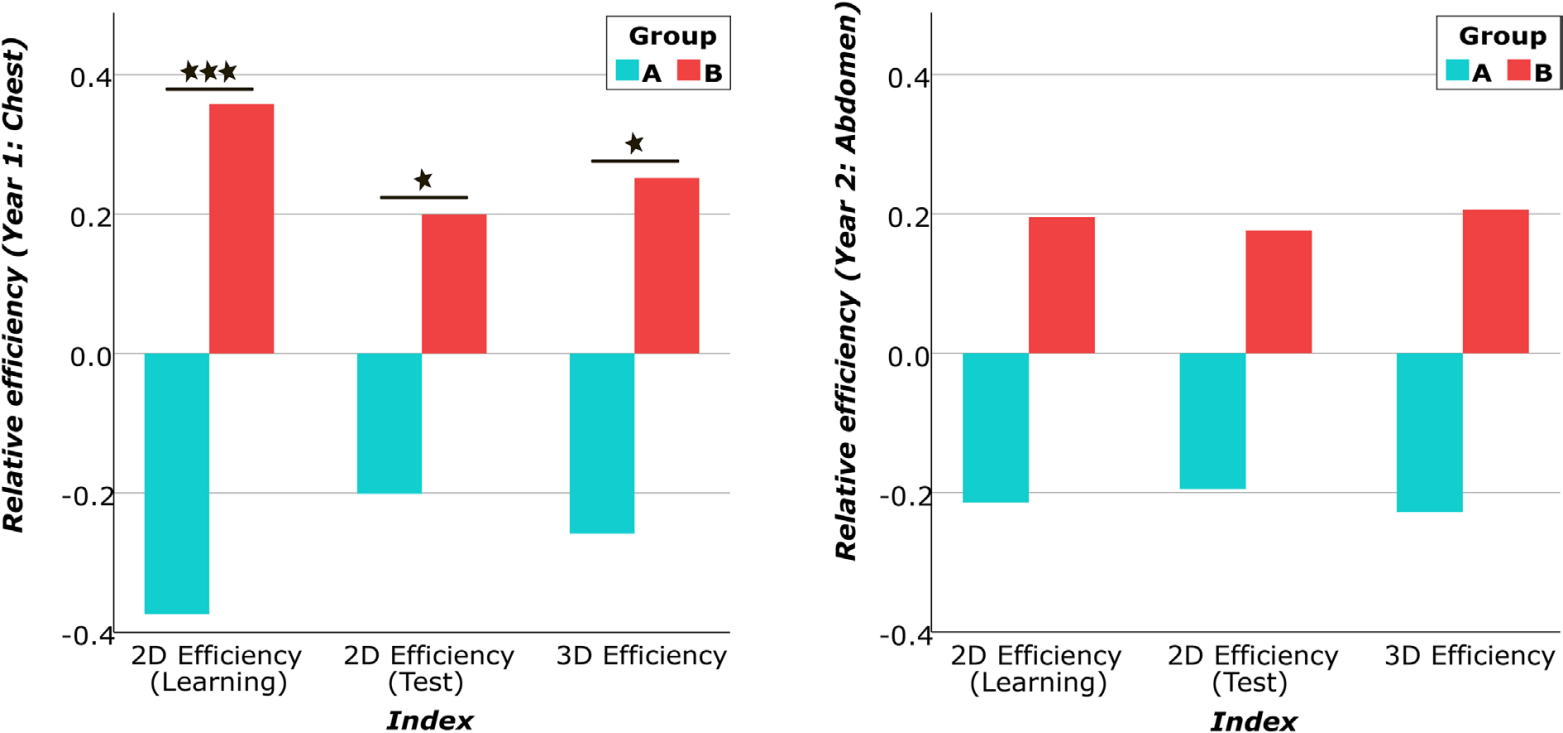
**Left graph:** Indices of relative instructional efficiency in Year 1 experiment. The 2D efficiency (L) index in Group A (mean = −0.37; SD = 1.15) was significantly lower (*t* = −3.631; *p* < 0.001) than in Group B (mean = 0.36; SD = 0.96). The 2D efficiency (T) index in Group A (mean = −0.20; SD = 1.05) was significantly lower (*t* = −2.066; *p* = 0.021) than in Group B (mean = 0.20; SD = 0.98). The 3D efficiency index in Group A (mean = −0.26; SD = 1.26) was significantly lower (*t* = −2.200; *p* = 0.015) than in Group B (mean = 0.25; SD = 1.17). **Right graph:** Indices of relative instructional efficiency in Year 2 experiment. The 2D efficiency (L) index in Group A (mean = −0.21; SD = 1.16) was lower (*t* = −1.367; *p* = 0.088) than in Group B (mean = 0.19; SD = 1.13). The 2D efficiency (T) index in Group A (mean = −0.19; SD = 1.04) was lower (*t* = −1.272; *p* = 0.104) than in Group B (mean = 0.18; SD = 1.18). The 3D efficiency index in Group A (mean = −0.23; SD = 1.24) was lower (*t* = −1.254; *p* = 0.107) than in Group B (mean = 0.21; SD = 1.39).

In both experiments, the MES showed that Groups A and B required progressively lower amounts of mental effort, from the pretest phase to the learning phase to the posttest phase. In both experiments, Group B required less mental effort than Group A in the learning phase (Figure 4). In both experiments, all computed indices of instructional efficiency were higher in Group B (Figure 5).

### Types of cognitive load

**FIGURE 6:**
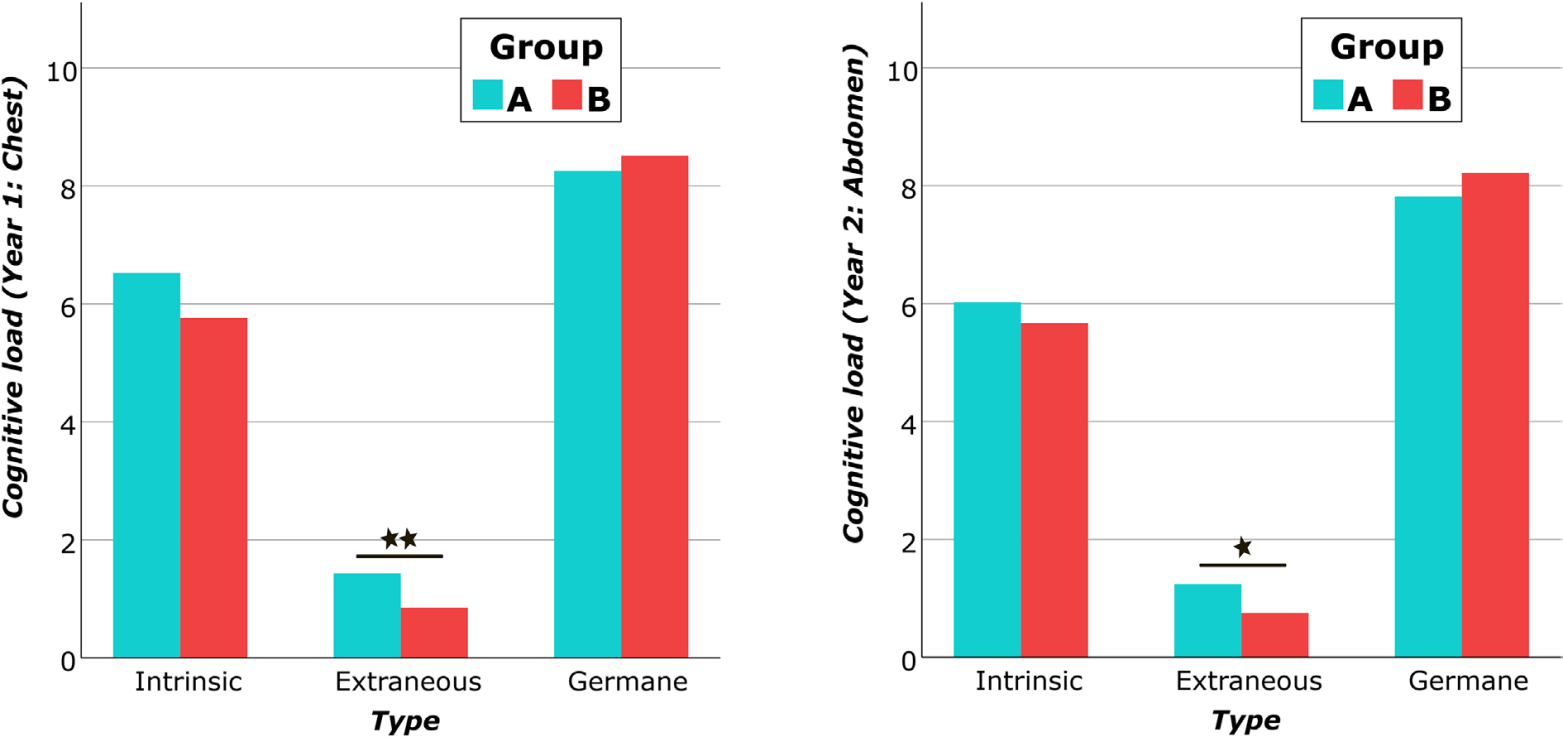
**Left graph:** Results of CLS in Year 1 experiment. Intrinsic load of Group A (mean = 6.52; SD = 1.61) was greater (*U* = 1231.000; *p* = 0.092) than Group B (mean = 5.76; SD = 2.28). Extraneous load of Group A (mean = 1.43; SD = 1.33) was significantly greater (*U* = 1079.000; *p* = 0.009) than Group B (mean = 0.85; SD = 1.05). Germane load of Group A (mean = 8.25; SD = 1.17) was lower (*U* = 1735.500; *p* = 0.180) than Group B (mean = 8.51; SD = 1.17). The intrinsic, extraneous, and germane load subscales of the CLS showed a Cronbach's alpha of 0.91, 0.87, and 0.93, respectively. **Right graph:** Results of CLS in Year 2 experiment. Intrinsic load of Group A (mean = 6.02; SD = 1.34) was greater (*t* = 0.859; *p* = 0.197) than Group B (mean = 5.67; SD = 1.80). Extraneous load of Group A (mean = 1.24; SD = 1.15) was significantly greater (*U* = 294.000; *p* = 0.031) than Group B (mean = 0.75; SD = 0.98). Germane load of Group A (mean = 7.81; SD = 1.43) was lower (*U* = 494.500; *p* = 0.357) than Group B (mean = 8.22; SD = 1.07). The intrinsic, extraneous, and germane load subscales of the CLS showed a Cronbach's alpha of 0.80, 0.74, and 0.92, respectively.

In both experiments, the CLS showed that Group B experienced less intrinsic cognitive load, significantly less extraneous cognitive load, and more germane cognitive load than Group A (Figure 6).

### Situational motivation and self‐determination

**FIGURE 7:**
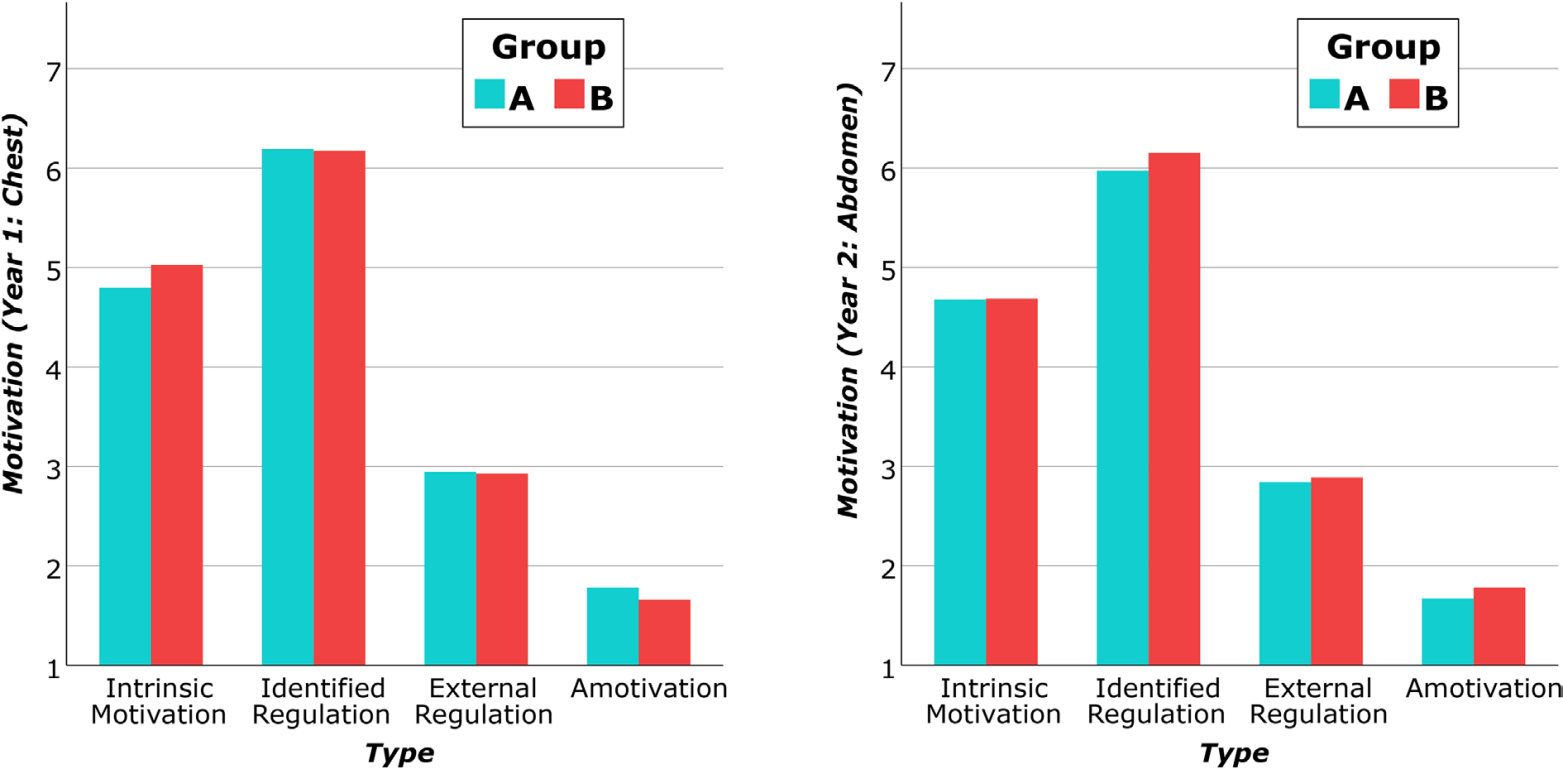
**Left graph:** Results of SIMS in Year 1 experiment. Intrinsic motivation of Group A (mean = 4.80; SD = 1.04) was lower (*t* = −1.1150; *p* = 0.126) than Group B (mean = 5.03; SD = 1.06). Identified regulation of Group A (mean = 6.19; SD = 0.63) was greater (*U* = 1529.500; *p* = 0.916) than Group B (mean = 6.17; SD = 0.75). External regulation of Group A (mean = 2.94; SD = 1.23) was greater (*U* = 1514.000; *p* = 0.990) than Group B (mean = 2.93; SD = 1.26). Amotivation of Group A (mean = 1.78; SD = 1.00) was greater (*U* = 1495.500; *p* = 0.920) than Group B (mean = 1.66; SD = 0.78). The self‐determination index of Group A (mean = 9.27; SD = 4.06) was lower (*U* = 1693.500; *p* = 0.278) than Group B (mean = 9.98; SD = 3.75). The intrinsic motivation, identified regulation, external regulation, and amotivation subscales of the SIMS showed a Cronbach's alpha of 0.75, 0.70, 0.73, and 0.84, respectively. **Right graph:** Results of SIMS in Year 2 experiment. Intrinsic motivation of Group A (mean = 4.68; SD = 0.96) was lower (*U* = 445.500; *p* = 0.861) than Group B (mean = 4.69; SD = 1.36). Identified regulation of Group A (mean = 5.97; SD = 0.85) was lower (*U* = 485.500; *p* = 0.431) than Group B (mean = 6.15; SD = 0.72). External regulation of Group A (mean = 2.84; SD = 1.48) was lower (*U* = 435.500; *p* = 0.982) than Group B (mean = 2.89; SD = 1.45). Amotivation of Group A (mean = 1.67; SD = 0.73) was lower (*U* = 494.000; *p* = 0.356) than Group B (mean = 1.78; SD = 0.68). The self‐determination index of Group A (mean = 9.15; SD = 2.55) was greater (*U* = 423.000; *p* = 0.867) than Group B (mean = 9.07; SD = 3.68). The intrinsic motivation, identified regulation, external regulation, and amotivation subscales of the SIMS showed a Cronbach's alpha of 0.85, 0.72, 0.82, and 0.71, respectively.

In both experiments, the SIMS showed that participants of Groups A and B were primarily driven by their identified regulation, followed by intrinsic motivation, external regulation, and finally, amotivation (Figure 7). There were no significant intergroup differences in the measured types of motivation nor in the computed self‐determination index.

### Usability of educational technology

**FIGURE 8:**
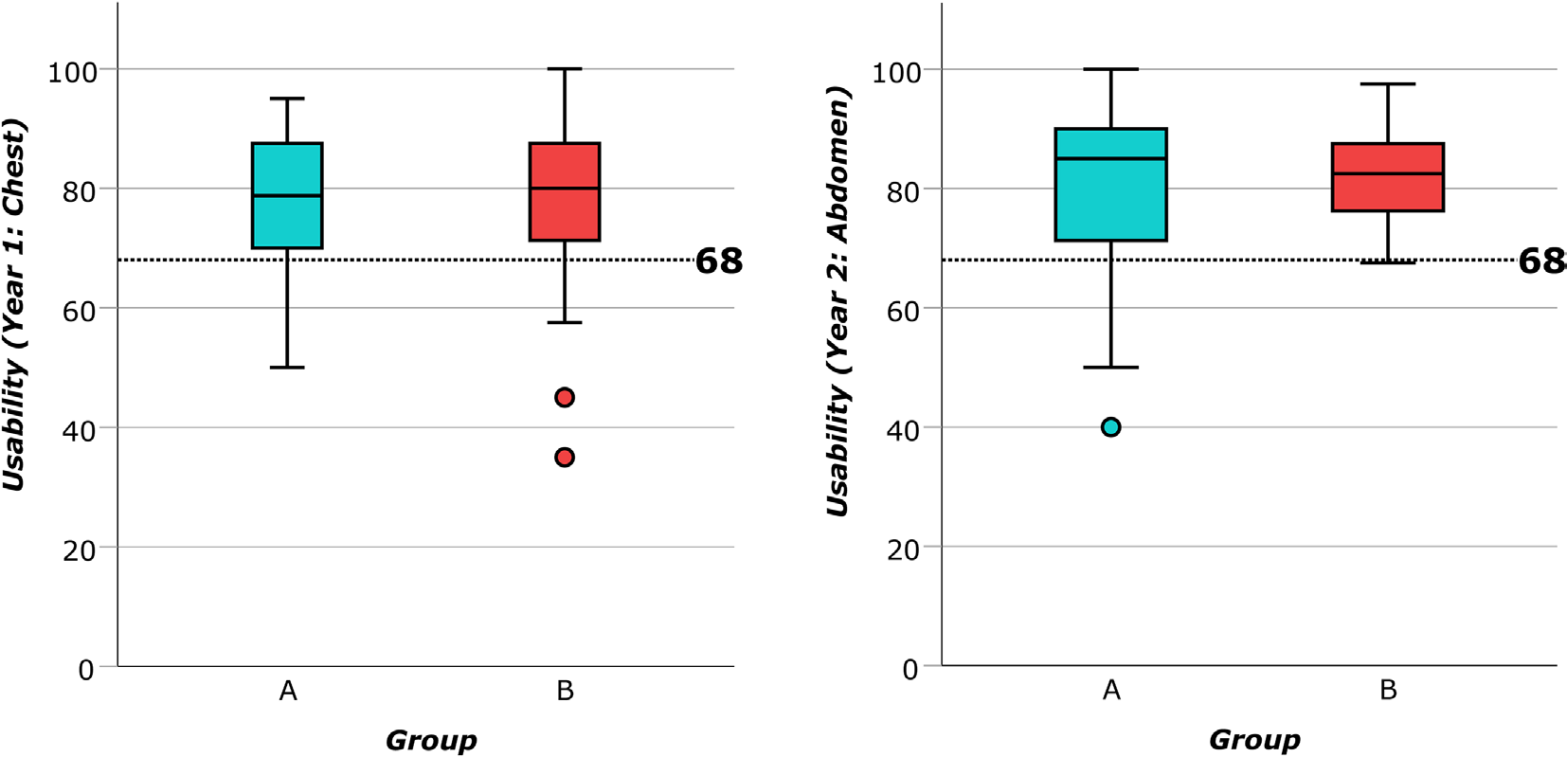
**Left graph:** Results of SUS in Year 1 experiment. Group A delivered a mean SUS score of 77.96 (SD = 12.06; min = 50.00; Q1 = 70.00; median = 78.75; Q3 = 87.50; max = 95.00). In comparison, Group B delivered a greater mean SUS score of 78.79 (SD = 12.96; min = 35.00; Q1 = 70.63; median = 80.00; Q3 = 87.50; max = 100.00). This difference was not significant (*U* = 1599.5; *p* = 0.600). The SUS revealed a Cronbach's alpha of 0.80. The dotted line indicates the threshold of usability (SUS = 68). **Right graph:** Results of SUS in Year 2 experiment. Group A delivered a mean SUS score of 80.54 (SD = 13.58; min = 40.00; *Q*1 = 70.63; median = 85.00; *Q*3 = 90.00; max = 100.00). In comparison, Group B delivered a greater mean SUS score of 81.94 (SD = 7.90; min = 67.50; *Q*1 = 75.00; median = 82.50; *Q*3 = 87.50; max = 97.50). This difference was not significant (*U* = 415.5; *p* = 0.778). The SUS revealed a Cronbach's alpha of 0.74. The dotted line indicates the threshold of usability (SUS = 68).

In both experiments, the SUS showed that participants of Groups A and B perceived the IMAIOS.com website as a usable resource for learning sectional anatomy (Figure 8). There were no significant intergroup differences in the perceptions of ed‐tech usability.

### Student perceptions of the learning activity

In the Year 1 chest‐focused experiment, 51 out of 54 participants in Group A (94%) and 54 out of 56 participants in Group B (96%) provided written feedback. In addition, in the Year 2 abdomen‐focused experiment, 26 out of 28 participants in Group A (93%), and 31 out of 31 participants in Group B (100%) provided written feedback. Five main themes were generated from the analysis of the aggregated written feedback provided by Year 1 and Year 2 participants.

#### Theme 1: Design and effectiveness of the learning session

Overall, “general feedback” showed that the session was well received despite some participants highlighting the “difficulty” of the learning experience. Nevertheless, many participants showed interest in attending “more sessions” focused on the sectional anatomy of other regions of the body. Participants emphasized that the “learning environment” and, especially, the “time” allocated for watching the video and using the website were major shaping factors in their learning experience. For example, some participants in both experiments, particularly of Group B, complained about the short duration of the learning activity.

The learning activity is challenging overall as I never had to interpret such images. —participant of Year 1, Group A

I am not a fast learner who remember things quickly. Hence, the time given for learning […] the sectional anatomy covered today was not sufficient to me. —participant of Year 1, Group B

More time could be provided for the learning activity, as the video takes up 20 minutes. I would be grateful for more time to explore the website. —participant of Year 2, Group B

#### Theme 2: Assessment, acquisition, and revision of knowledge

Many participants valued the session as an opportunity to “assess anatomical knowledge”. For example, some participants argued that the anatomy tests were important, not only in the context of the learning session itself, but also as a moment of formative assessment. Furthermore, the session was perceived as an opportunity to “revise and apply knowledge” obtained in lectures and dissections, and to “gain knowledge” about spatial anatomy, anatomical relationships, and CT images.

I liked that we went blind, learned how to read/understand the scans properly, before doing the questions again. It sorts of helps to better understand what's going on—by filling in gaps in knowledge. —participant of Year 1, Group A

I think it's interesting to use a lot of CT images to enhance my learning of [cardiopulmonary anatomy]. It combines knowledge gained from anatomy dissections and allowed me to put it in practice when going through the images. —participant of Year 1, Group A

I like the activity as an opportunity for me to revise the gastrointestinal anatomy, particularly the anatomical relationships between structures. —participant of Year 2, Group B

#### Theme 3: Effectiveness of the instructional video

The videos were praised for their “clarity and effectiveness”. Participants emphasized the importance of including “demonstrations” in the videos. In both experiments, some participants of Group A lamented the briefness of their video, which lacked any detailed anatomical explanations. Some participants provided suggestions for improving the videos.

Video was very clear. Watching the video before proceeding to the website help me gain a foundation beforehand. —participant of Year 2, Group B

The video was much more concise and informative, especially when dividing the structures by both topics (heart and lungs and chest) and levels (T1, T2, T3, etc). These helped me to integrate the anatomical and spatial knowledge together. —participant of Year 1, Group B

Maybe a bit more guidance on the spatial relationships of the vessels, and how to identify them. —participant of Year 2, Group A

#### Theme 4: Effectiveness of the learning website

Participants discussed, and mostly praised, the “usability” of the website (e.g., perceived ease of use and navigation) and the “interactivity” of the website (e.g., ability to scroll through the sectional images and select different annotations). Many participants commented on the “graphics and annotations” in the interface and highlighted the importance of the anatomical labels for learning. However, some participants criticized the complexity of the labeling system.

It was super helpful because I'm definitely more of a visual and practical learner so having a whole cross‐sectional image folder to scroll through on my own helped me visualize things better than textbooks and still images. —participant of Year 1, Group B

I personally like the pin function most since it can really test my anatomical knowledge efficiently at my own pace together with the video provided. —participant of Year 1, Group B

One thing that could improve is that sometimes the labels may be a bit too much, which may look a bit confusing and unfocused for some people. —participant of Year 1, Group B

#### Theme 5: Suggestions for future sessions

Participants suggested that future sessions should include “more multimodal” learning practices (e.g., incorporate more Netter images and 3D physical or digital models to complement the video and website), “more collaborative learning” activities (e.g., conducted with friends), and provide “more notes and handouts” to participants. Many participants displayed an interest in “accessing the learning materials”, both the video and website, for their own future use.

It is better to have a 3D model side‐by‐side with the CT scan for better visualization of the anatomical structures and to build a better spatial sense of what the CT image is referring to. —participant of Year 1, Group A

Collaboration would have made the learning easier and more engaging […]. Any confusion had to be figured out by myself, which wasn't always successful. —participant of Year 1, Group B

Can we get access to this website and video at home? I want to rewatch the video, it is quite useful. —participant of Year 1, Group B

## DISCUSSION

This study investigated the impact of incorporating audiovisual anatomical demonstrations (i.e., worked examples) in the technology‐enhanced instruction of sectional anatomy. The completion of two experiments with a pretest‐posttest design was fundamental for the acquisition of significant results. Furthermore, several factors support their validity. First, the stratified random sampling procedures generated equivalent groups at baseline in both the Year 1 and Year 2 experiments. In both experiments, Groups A and B displayed a similar gender distribution and proportion of participants with prior experience using the learning website IMAIOS.com (Table 1). Moreover, there were no significant intergroup differences in pretest knowledge, in either Year 1 or Year 2 experiments (Figure 3). Second, the experimental learning activities had a strict time limit, which was universally imposed on all participants. Third, the instructions for how to use the learning website IMAIOS.com and the anatomical demonstrations were provided in the form of standardized pre‐recorded videos. Fourth, all measurements conducted in this study relied on the use of valid and reliable instruments. Fifth and finally, similar patterns of results were found in both the Year 1 and Year 2 experiments conducted in this study.

### Hypothesis testing

Overall, the two experiments were adequate to test the hypotheses forwarded at the beginning of this study.

First, the results support *H1: The integration of worked examples in the technology‐enhanced learning of sectional anatomy increases knowledge gains*. In both experiments, the absolute and normalized knowledge gains were higher in Group B than in Group A. Furthermore, in the Year 1 experiment, the posttest knowledge scores of Group B were significantly higher than those of Group A.

Second, the results support *H2: The integration of worked examples in the technology‐enhanced learning of sectional anatomy lowers extraneous cognitive load*. In both experiments, Group B experienced significantly less extraneous cognitive load than Group A.

Third, and finally, the results also support *H3: The integration of worked examples in the technology‐enhanced learning of sectional anatomy increases instructional efficiency*. In both experiments, the three calculated indices of instructional efficiency were higher in Group B than in Group A. These intergroup differences were significant in the Year 1 experiment.

### Implications of the study for anatomy education

First, this study shows that outcome‐based anatomical demonstrations can improve the technology‐enhanced learning of sectional anatomy. In both experiments, participants of Group B, who had to invest time in observing and listening to an anatomical demonstration before using the website by themselves, gained more knowledge while experiencing less extraneous cognitive load and less overall mental effort in the learning phase (Figures 3, 4, 5, 6). These datapoints suggest a more effective and efficient learning experience. According to CLT, the teaching and learning methods should match the learner's level of expertise with the learning contents.^28^ Active learning strategies that incorporate student collaboration, problem‐solving, and hands‐on tasks are beneficial, if not essential, in anatomy education.^88^, ^89^ Nevertheless, the simple act of attentive thinking, which can be linked to the appropriate use of working memory, may define active learning by itself.^90^, ^91^ Moreover, the appropriate use of working memory is an essential step for learning, according to information processing frameworks that lie at the foundation of CLT and CTML.^92^ Therefore, whereas advanced learners may benefit more from exploratory and problem‐based tasks, novice learners, namely the participants of this study, may initially require more direct and explicit forms of instruction, namely a worked example, to guide their attention and help organize their thinking process.^28^ Notably, in both experiments, Group B also reported less intrinsic cognitive load and more germane cognitive load than Group A (Figure 6). Possibly, the anatomical demonstrations allowed participants of Group B to perceive (1) the learning contents to be less difficult or better scaffolded because the worked examples were helpful in increasing the relevant foundational knowledge of learners; and (2) the learning activity to be more productive or more engaging because the worked examples were helpful in demystifying, at a metacognitive level, the process of how to learn sectional anatomy. However, these interpretations are not definitive since these intergroup differences were non‐significant (Figure 6). Alternatively, it could be reasonably argued that the lower intrinsic cognitive load reported by Group B in both experiments, as assessed via the adapted CLS, actually translates to the learner perception that the demonstrations were effective in unraveling the learning contents. Theoretically, such perception would still correspond to a lower extraneous cognitive load, but not necessarily to a lower intrinsic cognitive load since the learning contents and their degree of inherent complexity were the same for Groups A and B. Fundamentally, the subjective nature of the CLS, combined with its short length (10 items), may not allow a fully discriminated quantification of intrinsic, extraneous, and germane cognitive loads. Unsurprisingly, issues related to the measurement of cognitive load have been a central topic of discussion and exploration within the fields of CLT and CTML.^64^, ^93^, ^94^ Lastly, the demonstrations were beneficial for learning, even though all participants had attended relevant lectures in anatomy and radiology and completed relevant dissections prior to the experimental learning sessions. This shows that the demonstrations were still appropriate for the participants' level of prior knowledge.^28^, ^95^ Anatomy teachers must carefully gauge, and not under/overestimate, the level of their students when providing a demonstration.

Second, this study provides additional support for the use of CLT and CTML in anatomy education. Several researchers have relied on these two learning theories to suggest the design, guide the delivery and/or evaluate the efficacy of a wide spectrum of teaching methods and materials in anatomy.^44^, ^55^, ^56^, ^57^, ^58^, ^59^, ^66^, ^96^, ^97^, ^98^, ^99^, ^100^, ^101^, ^102^, ^103^ In the current study, as predicted in CLT,^31^, ^33^ the inclusion of anatomical demonstrations generated a beneficial worked‐example effect in novice learners. While participants of Group A were only provided with a list of structures to identify in a CT scan of IMAIOS.com (i.e., the problem), participants of Group B were explicitly shown how to identify those structures in an organized fashion (i.e., the solution and the steps to understand it), before exploring the CT scan by themselves. Furthermore, as predicted in CTML,^26^ the demonstrations were effective because several principles of instructional design were intentionally applied during their recording, such as the modality principle (i.e., using spoken words to deliver information, instead of using solely the graphical elements of the website); the segmenting principle (i.e., dividing the demonstrations into logically arranged chapters); the signaling principle (i.e., using the mouse cursor to guide the attention of the learner); the redundancy principle (i.e., not using redundant labels and annotations available in the interface); and the coherence principle (i.e., focusing the demonstration on the learning outcomes). The signaling with the mouse cursor, combined with time‐congruent narrated explanations, may have been particularly important for learning. These factors enabled participants to learn the location and relationships of structures in the sectional images without having to rely on their own ability to find those structures or on the labeling system of the website, which was described by some as overwhelming. Research has shown that the analytical gaze of anatomy learners may depend on their spatial abilities and prior knowledge in anatomy.^104^, ^105^ Moreover, effective signaling in teaching may help novice learners to navigate tasks with a large quantity of visual information and may facilitate the initial learning of spatial anatomy.^96^, ^106^, ^107^

Third, the results suggest that the integration of preceding demonstrations did not have a significant impact on motivation, and vice versa (Figure 7). Even though the theoretical scope of CLT and CTML does not normally extend into non‐cognitive functions, it is important to understand how instructional conditions impact motivational processes. For instance, teaching and learning activities that impose unnecessary cognitive load may also increase the frustration and disengagement of students.^108^, ^109^ Consequently, excessive extraneous cognitive load in learning deteriorates student motivation, whereas reducing it may maintain or even increase motivation.^108^, ^109^ Students who are more motivated to learn, particularly those with more intrinsic motivation and overall self‐determination, tend to achieve better academic results.^78^ In the current study, measurements with the SIMS indicated that students' participation was driven primarily by their identified regulation, followed by their intrinsic motivation. According to Self‐Determination Theory,^75^ intrinsically motivated behaviors are conducted because of one's interest, enjoyment, and sense of self‐efficacy. In contrast, extrinsically motivated behaviors are conducted to attain external rewards, avoid punishment, or to achieve valued outcomes. And more specifically, extrinsically motivated behaviors that are more internally regulated (e.g., identified regulation) are performed because they yield outcomes that are personally valued. Thus, the results obtained with the SIMS seem consistent with the written feedback of participants. For example, while many participants revealed that they found the experimental learning activity to be interesting and enjoyable (related to intrinsic motivation), many also commented on the importance of the activity as an opportunity to obtain, assess, and apply anatomical knowledge (related to identified regulation). Furthermore, from a motivational perspective,^75^, ^108^ both instructional conditions, with and without demonstrations, appeared equally impactful as shown by the SIMS, because they likely generated enough engagement (e.g., participants controlled the use of the website) and provided enough structure (e.g., participants received guidance via the instructional videos) in learning, thus satisfying the participants' psychological needs of autonomy and competence.

Fourth, the results suggest that the website IMAIOS.com is a useful pedagogical resource in sectional anatomy (Figure 8). In both experiments, Groups A and B assessed the IMAIOS.com website with mean SUS scores always above 68, the threshold of usability.^81^, ^82^ These results match the generally positive written feedback provided by participants regarding the website. In addition, the lack of significant intergroup differences in either experiment shows that the inclusion of a demonstration did not have a significant impact on perceptions of IMAIOS.com usability, and vice versa. Nevertheless, and unsurprisingly, some participants in both experiments, mostly of Group B, suggested that more time should have been provided for exploring the website. Furthermore, it was interesting to detect that the mean SUS scores provided by the Groups involved in this study, ranging from 77.96 to 81.94, were all higher than the score of 67.64 provided by a group of 37 medical students who had used the same platform to learn sectional anatomy in a previous exploratory study.^44^ Although the website itself was updated in the time between studies, it could be assumed that the user experience was also influenced by the different pedagogical methods employed in these studies. For example, while participants in the current study used the website individually on a computer, participants in the aforementioned exploratory study had to work in small groups and use the website on a shared Microsoft Surface Hub. This observation reinforces the need for researchers investigating the impact of technology in anatomy learning to assess the usability of the educational technologies used while also detailing the specific instructional methods employed.^110^, ^111^

Fifth and finally, participants provided some ideas for improvements. Most notably, participants expressed that future sessions should be (1) more multimodal and (2) more collaborative. The first suggestion is compatible with the multiple representations principle of instructional design described in CTML^112^ and with published recommendations for anatomy teaching, including 2D sectional anatomy.^113^, ^114^ This suggestion also aligns with previous research showing that the examination of 3D whole anatomy with cadaveric dissection,^115^, ^116^, ^117^ digital models,^118^, ^119^, ^120^, ^121^ physical models,^5^, ^122^, ^123^ or even clay models^124^, ^125^ can support and facilitate the concurrent learning of relevant 2D sectional anatomy. Regarding the second suggestion, running a more collaborative learning activity would also better represent the cooperative pedagogical environment oftentimes observed in anatomy teaching laboratories.^89^ More importantly, well‐designed collaborative learning activities can potentially further enhance the motivation of learners because teamwork may satisfy their psychological need for relatedness.^75^ Similarly, collaboration may further improve the cognitive efficiency of learning.^126^, ^127^ Nevertheless, despite requests for increased multimodality and collaboration, the current study shows that instructional activities in sectional anatomy can be successfully completed by individual learners with just (1) SMART learning outcomes; (2) a usable visualization technology with dynamic and interactive sectional images; and (3) complementary static images from a popular anatomy atlas. Finally, the inclusion of (4) an outcome‐focused audiovisual anatomical demonstration may increase the efficacy and efficiency of learning among novices.

### Limitations

Six main limitations were identified in this study.

First, participation in the study was entirely voluntary. Thus, it is likely that the samples were mostly comprised of highly motivated and academically achieving students, inherently more interested in anatomy and in the contents of the learning activity.

Second, the instructional videos were not fully optimized. Although effective enough, the instructional videos, including the anatomical demonstrations, were far from perfect. These teaching materials, which were created and used for the first time in this study, can be improved. Ultimately, better demonstrations may generate even more impactful worked‐example effects in anatomy learning.

Third, the exact amount of time each participant invested in watching the instructional video or using the website was not measured. At the start of the learning phase, all participants had to play their respective video (Figure 2B). However, participants could then freely control it (e.g., going backward and forward or rewatching different sections). Therefore, this study could only control the total amount of time provided for the whole learning activity and the *theoretical* duration of the instructional videos.

Fourth, the anatomy tests were likely too easy in the Year 2 experiment because the mean pretest knowledge scores were relatively high (Figure 3). This latent ceiling effect impacted the subsequent calculation of knowledge gains and instructional efficiency. This prevented the attainment of results as significant as those verified in the Year 1 experiment, even though similar patterns of outcomes were found.

Fifth, the SIMS and the SUS were completed by participants only after the learning activity. Therefore, it is impossible to determine how motivation and perceptions of ed‐tech usability evolved from pretest to posttest without embracing a logical fallacy.

Sixth, and finally, the findings of this study represent short‐term effects. Longer‐term outcomes remain to be explored.

## CONCLUSION

In conclusion, this study supports the incorporation of explicit and outcome‐focused audiovisual anatomical demonstrations in the technology‐enhanced instruction of sectional anatomy for novice learners. In this study, first‐ and second‐year undergraduate medical students who watched an anatomical demonstration before exploring a visualization technology (IMAIOS.com) by themselves learned sectional anatomy more effectively and efficiently than peers who only used the visualization technology in a more self‐directed manner. Thus, a “demonstration before exploration” instructional approach appears more beneficial for novice students than a sole “exploration”. In addition, this approach does not appear to significantly impact student motivation nor perceptions of ed‐tech usability. Nevertheless, this teaching strategy may not be as effective if (1) the students are more knowledgeable beforehand, or if (2) the learning contents are intrinsically less difficult for students. Overall, the findings of this study are consistent with learning theories, namely CLT and CTML, which highlight the importance of including worked examples in the instruction of novice learners. We encourage other researchers to investigate both the short‐term and long‐term effects of worked examples in other contexts of anatomy education. Further research is also needed to explore which factors make an anatomical demonstration measurably beneficial for the understanding of spatial anatomy.

## AUTHOR CONTRIBUTIONS

**Fraide Augustin Ganotice Jr:** Writing – review and editing; conceptualization; methodology. **Guilherme Reis Borges Coelho Fonseca:** Conceptualization; writing – original draft; writing – review and editing; methodology; formal analysis; data curation; investigation. **George Lim Tipoe:** Writing – review and editing; conceptualization; methodology.

## Supporting information


Data S1.

Video S1.

## Data Availability

The data that support the findings of this study are available on request from the corresponding author. The data are not publicly available due to privacy or ethical restrictions.
