# Supplementary material for: Comparing the outcomes of the technology‐enhanced learning of sectional anatomy with and without preceding demonstrations: A study about the worked‐example effect in anatomy education
Source: Anat Sci Educ. 2026 May 25;19(9):1396–414. doi: 10.1002/ase.70267 (PMC13545339; doi:10.1002/ase.70267)
Supplement: Supplementary file 1 [file ASE-19-1396-s001.docx]

SUPPLEMENTARY MATERIAL

[Learning Outcomes (Year 1: Chest)	2](#_Toc203936863)

[Learning Outcomes (Year 2: Abdomen)	3](#_Toc203936864)

[Anatomy Test (Year 1: Chest)	4](#_Toc203936865)

[Anatomy Test (Year 2: Abdomen)	7](#_Toc203936866)

[Mental Effort Scale (MES)	10](#_Toc203936867)

[Cognitive Load Scale (CLS)	11](#_Toc203936868)

[Situational Motivation Scale (SIMS)	12](#_Toc203936869)

[System Usability Scale (SUS)	13](#_Toc203936870)

# Learning Outcomes (Year 1: Chest)

| **By the end of this activity, you should be able to recognize the following 35 structures in sectional images of the CHEST.** | |
| --- | --- |
| **Mediastinum**   - *Heart*   - Left atrium   - Right atrium   - Left ventricle   - Right ventricle - *Arteries*   - Pulmonary trunk   - Right pulmonary artery   - Left pulmonary artery   - Ascending aorta   - Arch of aorta   - Descending aorta   - Brachiocephalic trunk   - Left common carotid artery   - Left subclavian artery - *Veins*   - Pulmonary veins   - Superior Vena Cava   - Azygos vein   - Left brachiocephalic vein   - Right brachiocephalic vein - *Other structures*   - Trachea   - Right main bronchus   - Left main bronchus   - Esophagus | **Lungs**   - *Right lung*   - Superior lobe   - Middle lobe   - Inferior lobe - *Left lung* - Superior lobe - Inferior lobe   **Walls of chest**   - *Bones*   - Vertebrae T1 to T12   - Ribs   - Sternum - *Muscles*   - Diaphragm   - Pectoralis major   - Pectoralis minor   - Intercostal muscles   - Serratus anterior |

# Learning Outcomes (Year 2: Abdomen)

| **By the end of this session, you should be able to recognize the following 36 structures in sectional images of the ABDOMEN.** | |
| --- | --- |
| **Viscera of abdomen**   - *Major viscera*   - Liver   - Gallbladder   - Spleen   - Pancreas   - Kidneys   - Suprarenal glands - *Gastrointestinal tract*   - Stomach   - Duodenum   - Jejunum and Ileum   - Caecum   - Ascending colon   - Transverse colon   - Descending colon | **Vascular structures of abdomen**   - *Arteries*   - Abdominal aorta   - Right renal artery   - Left renal artery   - Celiac trunk   - Common hepatic artery   - Left gastric artery   - Splenic artery   - Superior mesenteric artery   - Inferior mesenteric artery - *Veins*   - Inferior vena cava   - Right renal vein   - Left renal vein   - Hepatic veins   - Portal vein   - Splenic vein   - Superior mesenteric vein   **Walls of abdomen**   - *Vertebral column* - *Muscles*   - Diaphragm   - Rectus abdominis   - Transversus abdominis   - Internal oblique   - External oblique   - Psoas major   - Quadratus lumborum |

# Anatomy Test (Year 1: Chest)

**The CT images used in the test were adapted from multiple textbooks in sectional and radiological anatomy (Currie et al., 2009; Ellis et al., 2015; Herring, 2019; Kelley & Petersen, 2018; Spratt et al., 2020).**

# Anatomy Test (Year 2: Abdomen)

**The CT images used in the test were adapted from multiple textbooks in sectional and radiological anatomy (Currie et al., 2009; Ellis et al., 2015; Herring, 2019; Kelley & Petersen, 2018; Spratt et al., 2020).**

# Mental Effort Scale (MES)

**AFTER PRETEST**, rate your **mental effort** required to complete the first test (encircle a number).

**AFTER LEARNING ACTIVITY**, rate your **mental effort** required to complete the learning activity (encircle a number).

**AFTER POSTTEST**, rate your **mental effort** required to complete the second test (encircle a number).

# Cognitive Load Scale (CLS)

All the following statements refer to the learning activity (*not the tests*) that just finished. Read each of the statements carefully and respond to each of them on a scale from 0 to 10 (encircle a number), in which “**0**” indicates ***not at all the case***; and “**10**” indicates ***completely the case***:

*Not at all the case*

*Completely the case*

| 1. The topics covered during the activity were very complex. | 0 | 1 | 2 | 3 | 4 | 5 | 6 | 7 | 8 | 9 | 10 |
| --- | --- | --- | --- | --- | --- | --- | --- | --- | --- | --- | --- |
| 2. The activity covered anatomical images that I perceived as very complex. | 0 | 1 | 2 | 3 | 4 | 5 | 6 | 7 | 8 | 9 | 10 |
| 3. The activity covered anatomical structures and spatial relationships that I perceived as very complex. | 0 | 1 | 2 | 3 | 4 | 5 | 6 | 7 | 8 | 9 | 10 |
| 4. The instructions and learning materials during the activity were very unclear. | 0 | 1 | 2 | 3 | 4 | 5 | 6 | 7 | 8 | 9 | 10 |
| 5. The instructions and learning materials during the activity were full of unclear language and confusing visual elements. | 0 | 1 | 2 | 3 | 4 | 5 | 6 | 7 | 8 | 9 | 10 |
| 6. The instructions and learning materials during the activity were, in terms of learning, very ineffective. | 0 | 1 | 2 | 3 | 4 | 5 | 6 | 7 | 8 | 9 | 10 |
| 7. The activity really enhanced my understanding of the topics covered. | 0 | 1 | 2 | 3 | 4 | 5 | 6 | 7 | 8 | 9 | 10 |
| 8. The activity really enhanced my understanding of the anatomical images covered. | 0 | 1 | 2 | 3 | 4 | 5 | 6 | 7 | 8 | 9 | 10 |
| 9. The activity really enhanced my knowledge of anatomical structures and spatial relationships. | 0 | 1 | 2 | 3 | 4 | 5 | 6 | 7 | 8 | 9 | 10 |
| 10. The activity really enhanced my understanding of sectional anatomy. | 0 | 1 | 2 | 3 | 4 | 5 | 6 | 7 | 8 | 9 | 10 |

# Situational Motivation Scale (SIMS)

Read each item carefully. Using the scale below, please circle the number that best describes the reason why you engaged in this activity. Answer each item according to the following scale: **1: corresponds not at all**; **2: corresponds very little**; **3: corresponds a little**; **4: corresponds moderately**; **5: corresponds enough**; **6: corresponds a lot**; **7: corresponds exactly.**

*Corresponds not at all*

*Corresponds exactly*

| 1. Because I thought that the activity was interesting | 1 | 2 | 3 | 4 | 5 | 6 | 7 |
| --- | --- | --- | --- | --- | --- | --- | --- |
| 2. Because I was doing it for my own good | 1 | 2 | 3 | 4 | 5 | 6 | 7 |
| 3. Because I was supposed to do it | 1 | 2 | 3 | 4 | 5 | 6 | 7 |
| 4. There may have been good reasons to do this activity, but personally I don’t see any | 1 | 2 | 3 | 4 | 5 | 6 | 7 |
| 5. Because I thought that this activity was pleasant | 1 | 2 | 3 | 4 | 5 | 6 | 7 |
| 6. Because I thought that this activity was good for me | 1 | 2 | 3 | 4 | 5 | 6 | 7 |
| 7. Because it was something that I had to do | 1 | 2 | 3 | 4 | 5 | 6 | 7 |
| 8. I did this activity, but I am not sure if it was worth it | 1 | 2 | 3 | 4 | 5 | 6 | 7 |
| 9. Because this activity was fun | 1 | 2 | 3 | 4 | 5 | 6 | 7 |
| 10. By personal decision | 1 | 2 | 3 | 4 | 5 | 6 | 7 |
| 11. Because I didn’t have any choice | 1 | 2 | 3 | 4 | 5 | 6 | 7 |
| 12. I don’t know; I don’t see what this activity brought me | 1 | 2 | 3 | 4 | 5 | 6 | 7 |
| 13. Because I felt good when doing this activity | 1 | 2 | 3 | 4 | 5 | 6 | 7 |
| 14. Because I believe that this activity was important for me | 1 | 2 | 3 | 4 | 5 | 6 | 7 |
| 15. Because I feel that I had to do it | 1 | 2 | 3 | 4 | 5 | 6 | 7 |
| 16. I did this activity, but I am not sure it was a good thing to pursue it | 1 | 2 | 3 | 4 | 5 | 6 | 7 |

# System Usability Scale (SUS)

Rate your level of agreement with each statement in the scale below.

The *system* mentioned in the items of the scale is the website **IMAIOS.com.**
